# Editorial: Therapeutic Implications for Pregnant Women With Systemic Autoimmune Diseases and Their Children

**DOI:** 10.3389/fphar.2021.773970

**Published:** 2021-11-26

**Authors:** Cecilia Beatrice Chighizola, Guilherme Ramires de Jesús, Maria Gerosa, Tadej Avčin

**Affiliations:** ^1^ Department of Clinical Sciences and Community Health, Research Center for Adult and Pediatric Rheumatic Diseases, University of Milan, Milan, Italy; ^2^ Pediatric Rheumatology Unit, ASST G. Pini & CTO, Milan, Italy; ^3^ Department of Obstetrics, Instituto Fernandes Figueira, FIOCRUZ, Rio de Janeiro, Brazil; ^4^ Universidade do Estado do Rio de Janeiro, Rio de Janeiro, Brazil; ^5^ Clinical Rheumatology Unit, ASST G. Pini & CTO, Milan, Italy; ^6^ Department of Allergology, Rheumatology and Clinical Immunology, Children’s Hospital, Ljubljana, Slovenia; ^7^ University Medical Centre Ljubljana, University of Ljubljana, Ljubljana, Slovenia

**Keywords:** pregnancy, rheumatic diseases, treatment, obstetric complications, disease activity, offspring

The close interplay between systemic autoimmune diseases and reproductive health has long been known: gestations in women with rheumatic disease are at higher risk of maternal and fetal complications. In addition, the physiologic hormonal changes that characterize the different phases of pregnancy modulate disease activity, in some diseases exacerbating the disease activity, while in others improving the disease severity. The impact of disease activity on reproductive issues in rheumatology is crucial: in many different conditions, disease activity before and during pregnancy is the main predictor of gestational outcome. This observation implies the compelling relevance of tight disease control throughout gestational course, which in turn translates into the necessity to ameliorate the pharmacological approach to rheumatic pregnant patients.

If the impact of rheumatologic conditions on pregnancy progression had been ascertained in the early days, the scientific community could not yet reach a unanimous consensus whether rheumatic conditions can affect female fertility. As a matter of fact, women with rheumatoid arthritis (RA), the most common rheumatologic inflammatory disease, take a longer time to conceive and have decreased family size compared to healthy controls. Again, active disease is the main determinant of these difficulties in achieving conception, but the exact mechanisms underlying such subfertility are still uncharacterized. Bongenaar et al. investigated the potential role in RA-associated female subfertility of interleukin (IL)-6 and tumour necrosis factor (TNF)-α, two pro-inflammatory mediators exerting a key pathogenic role. In a cohort of 61 RA women seeking a pregnancy, preconceptional IL-6 but not TNF-α levels predicted prolonged time to pregnancy, an association that held significance even after correction for confounders as disease activity.

The pivotal importance of optimal disease control reflects on the requirement of valid tools to evaluate disease activity even during pregnancy. Unfortunately, the reliability of conventional measures of disease activity in pregnant women is to be assessed: gestation may induce symptoms evaluated by these instruments and the physiological changes that occur in pregnancy may affect laboratory parameters used to assess disease activity, such as serum complement levels and C reactive protein (Andreoli et al., 2019). According to the data presented by Larosa et al., SLE disease activity score (SLE-DAS), a score composed by 17 items that has been recently proposed in SLE (Jesus et al., 2019), is a reliable tool to evaluate disease activity in pregnant lupus patients. SLE-DAS evaluated in 158 SLE women during the first trimester of gestation predicted maternal flares in the second and third trimesters (Larosa et al.).

Similarly to SLE, autoimmune bullous diseases can manifest or exacerbate during pregnancy, as elegantly reviewed by the group of Marzano et al. The pharmacological approach to pregnant women with pemphigus and pemphigoid gestationis is challenging, and the therapeutic armamentarium includes systemic and topical corticosteroids, topical calcineurin inhibitors, dapsone, intravenous immunoglobulins, plasmapheresis/plasma exchange and biological agents.

The introduction of biologics has revolutionized the management of rheumatic diseases over the last decades; moreover, insights are being progressively gained on their use in pregnant women. Given their immunoglobulinic structure, biologics can cross the placenta from the 17th gestational week, when the Fc receptor becomes expressed. As outlined by Beltagy et al., inhibitors of TNF-α (in particular certolizumab and etanercept, as well as eculizumab, due to its engineered IgG2/4k molecular structure) are considered safe during gestational course. Carnovale et al. focused on agents targeting IL-1, together with colchicine, estimating maternal and fetal adverse events in a nested case-control study on data from the United States Food and Drug Administration Adverse Event Reporting System database. Reassuringly, the rates of pregnancy loss, fetal disorders, congenital defects, neonatal adverse events and delivery complications were not increased in women receiving inflammasome-targeted therapy (Carnovale et al.).

In women with rheumatic diseases, the above-cited Fc receptor mediates even the active transportation of maternal autoantibodies, some of which can induce a passively acquired autoimmune disease. This is the case of neonatal anti-phospholipid syndrome, an exceedingly rare condition in newborns. Paediatric rheumatologists describe four cases of stroke in neonates born to women with anti-phospholipid antibodies (Giani et al.). Three out of the four children had positive anti-ß2GPI and aCL IgG, which disappeared within 2 years of life and could be due to transplacental autoantibody transfer. One baby presented anti-ß2GPI IgM, while her mother tested negative for all aPL subtypes suggesting neonatal *de novo* production of aPL. The exact pathogenetic role of transmitted or *de novo* aPL in neonatal ischemic stroke is still unclear and may be linked with other genetic and acquired thrombophilic factors. Surely pharmacological treatment of rheumatic conditions in pregnant women allows a tighter disease control and, in turn, better obstetric and fetal outcomes. However, concerns have arisen about the potential long-term effects of *in utero* exposure to anti-rheumatic medications. A multi-disciplinary team of rheumatologists and child neuropsychiatrists investigated the impact of treatment during gestation in 18 school-age children born to 16 women with inflammatory arthritis. Neurodevelopmental issues were found to be more frequent in the offspring of patients with longer disease duration and those who were breastfed for less than 6 months (Nalli et al.). Lazzaroni et al. focused on the neuropsychiatric impact of exposure to azathioprine during fetal life in children born to women with SLE. In this prospective study, offspring of lupus patients had a similar neuropsychiatric outcome compared to the general pediatric population, and no difference emerged stratifying upon *in utero* exposure to azathioprine (Lazzaroni et al.).

These data are reassuring, further supporting the importance of appropriate prescription of available anti-rheumatic drugs to obtain disease control in pregnant patients. However, research efforts are still pursued to identify novel pharmacological targets. Yao et al. reviewed the relationship between maternal microbiome and obstetric complications such as preeclampsia. The microbiome in the mother’s vagina and breast milk can also affect the development of neonatal nervous and immune systems (Yao et al.). Interestingly, not only gut microbiome undergoes several modifications during pregnancy and lactation, but also several pharmacological compounds commonly used in the treatment of inflammatory arthritis affect gut microbiome. Thus, as proposed by a Chinese group, the modulation of gut microbiome might affect disease activity in pregnant women with RA: prebiotics and probiotics, antibiotics, fecal microbiota transplantation and dietotherapy are among the potential strategies (Yao et al.).

As a whole, the manuscripts enclosed in the present research topic clearly highlight how rapidly the field is emerging: the times in which women with rheumatic diseases were discouraged from embarking in a pregnancy are luckily far away. Nevertheless, despite the much recent advancement, there are still many issues that should be unraveled to further improve the clinical management and the therapeutic approach to mothers-to-be as well as new mothers with rheumatologic conditions and their offspring.
